# Supplementary material for: Genomic characterization of Lao Mobatvirus strains reveals host-associated clustering in Southeast Asian bats
Source: J Gen Virol. 2026 Sep 9;107(9):002333. doi: 10.1099/jgv.0.002333 (PMC13557741; doi:10.1099/jgv.0.002333)

Figure S1. Sankey diagram summarizing number of animals captured and surveyed with taxonomic information. The total counts are aggregated from live-captured bats and wild animals surveyed in local wet markets (Chiroptera, Rodentia, Primates, Carnivora, and Scandentia). The diagram flows from total samples -> order -> family -> genus -> species

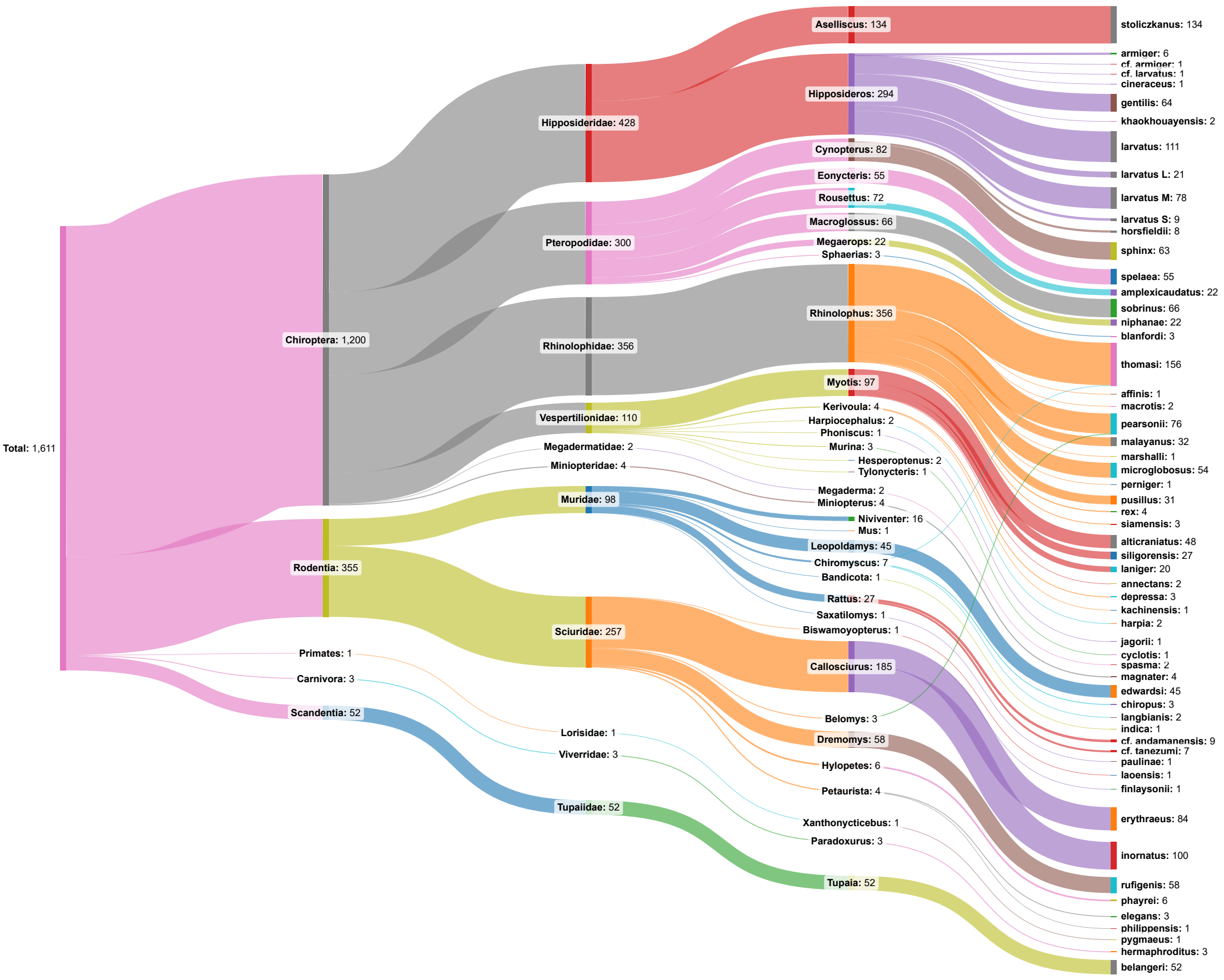

Figure S2. Pie chart showing the composition of the screened samples. Slices represent sample types with counts indicated for each slice.

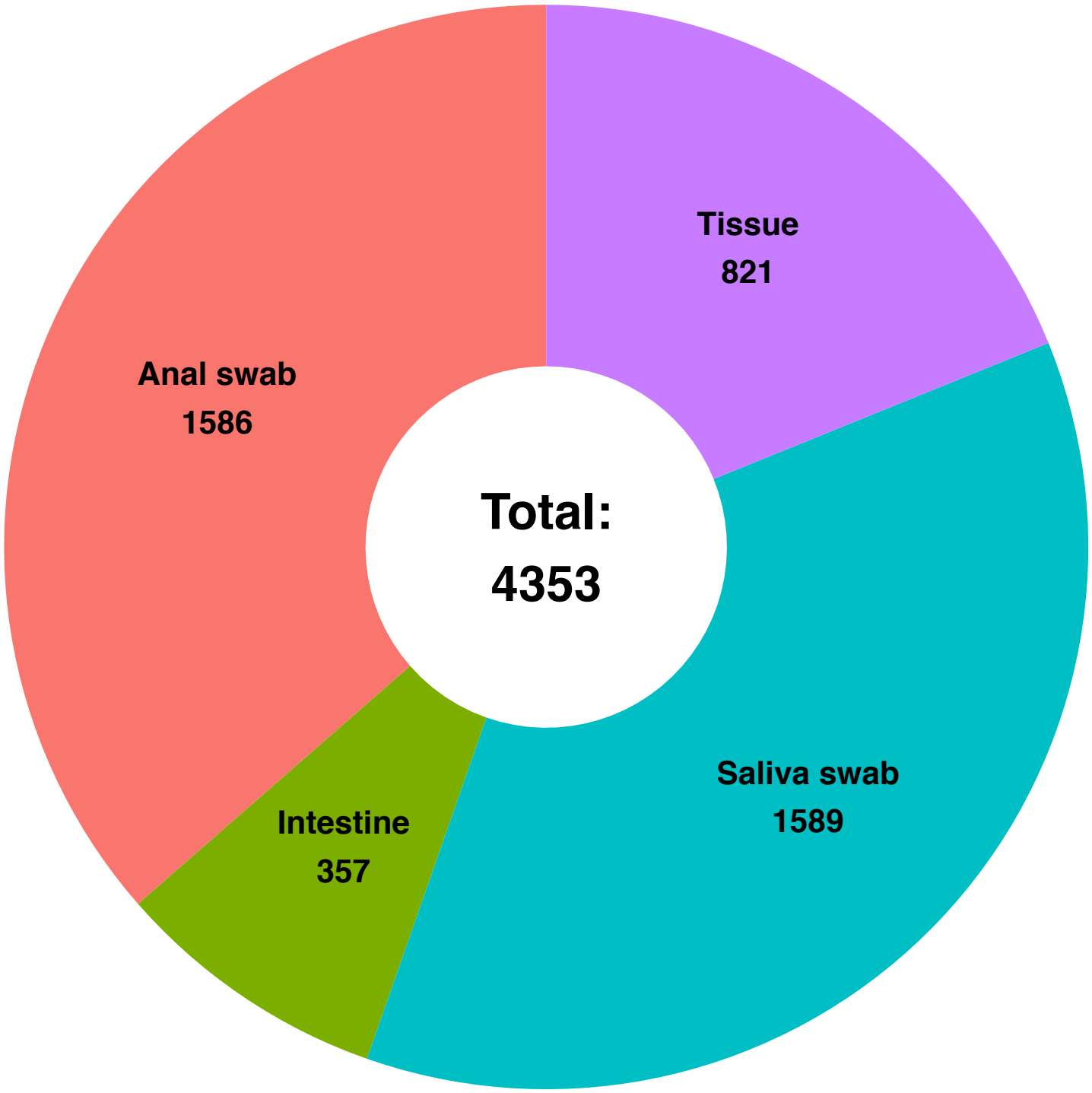

Figure S3. Comparison of predicted transmembrane structure of glycoprotein for LBHV and representative hantavirids. Representative hantavirus sequences used for comparison are listed in Table S3.

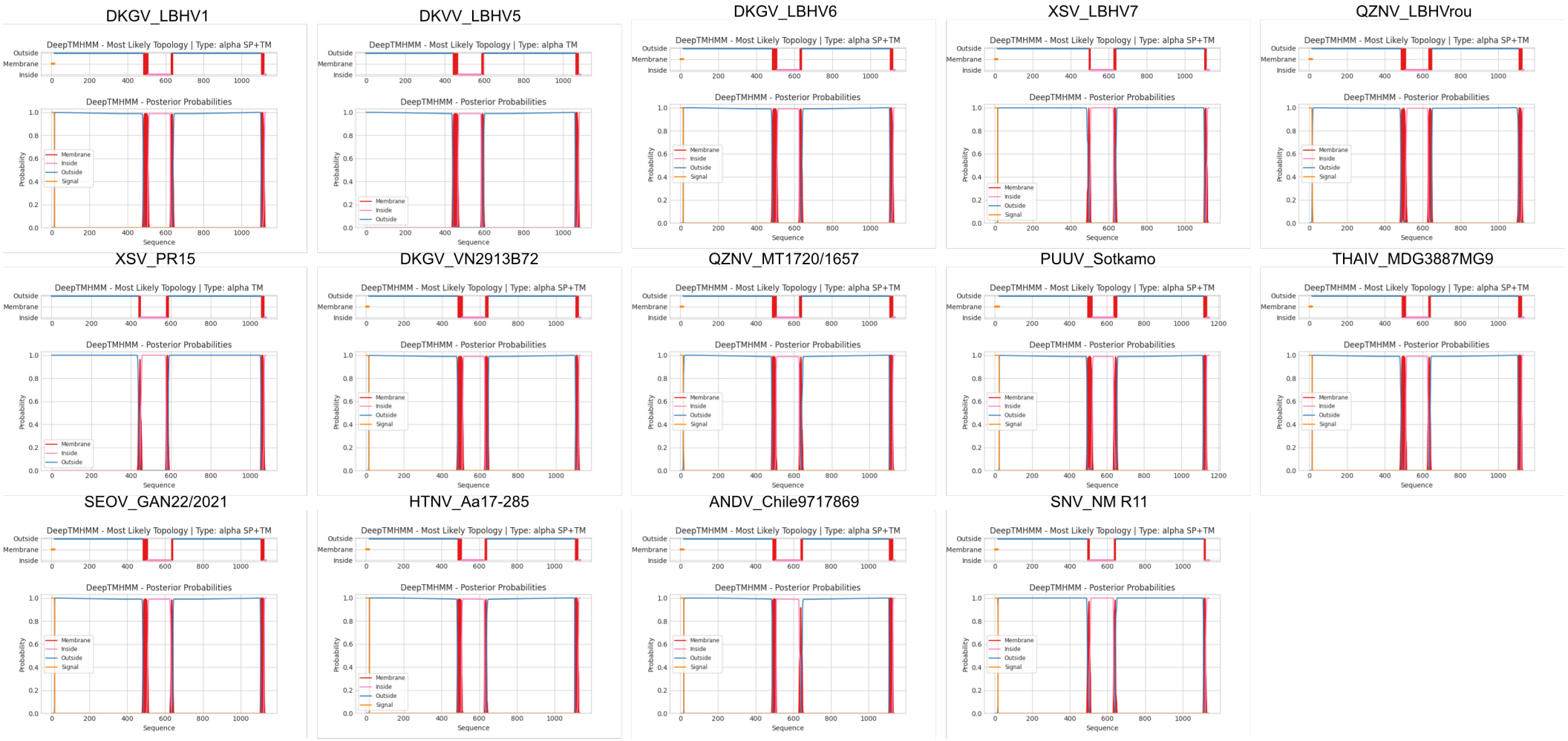

Figure S4. Predicted secondary structure maps for the S and M segment proteins. Secondary structure elements were extracted from AlphaFold3-predicted models (see Methods and Table S2). Helices, sheets and coils are indicated by standard schematic symbols; conservation (% conserved)

M segment

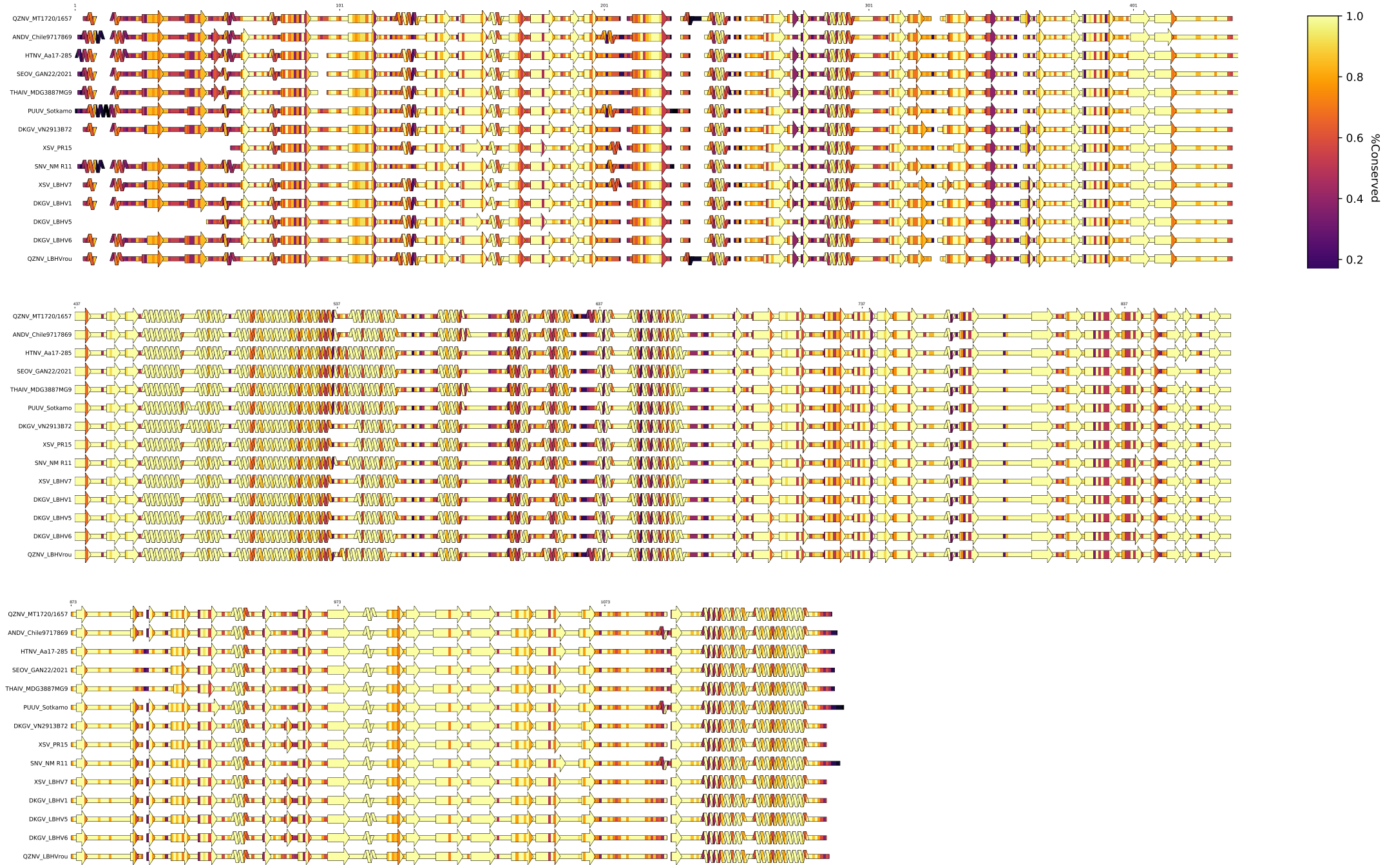

S segment

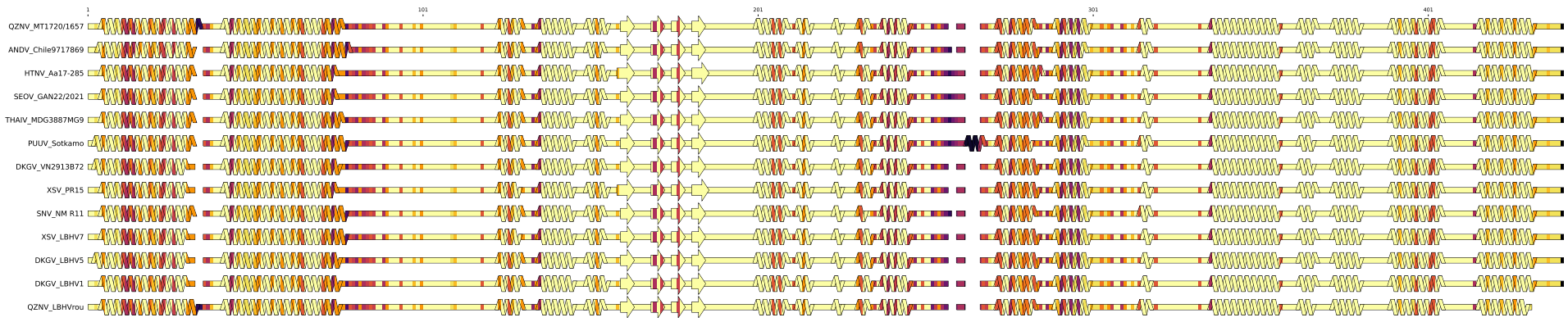

Figure S5. ML trees of both nucleotide (nt) and amino-acid (aa). LBHV sequences are highlighted in red, and red rectangles denote major clades. Blue triangle indicates rodent-borne orthohantaviruses. Scale bars indicate nucleotide substitutions per site for nucleotide trees and amino-acid substitutions per site for amino-acid trees. Sequence accession numbers and detailed information of abbreviated tip names are provided in Table S3

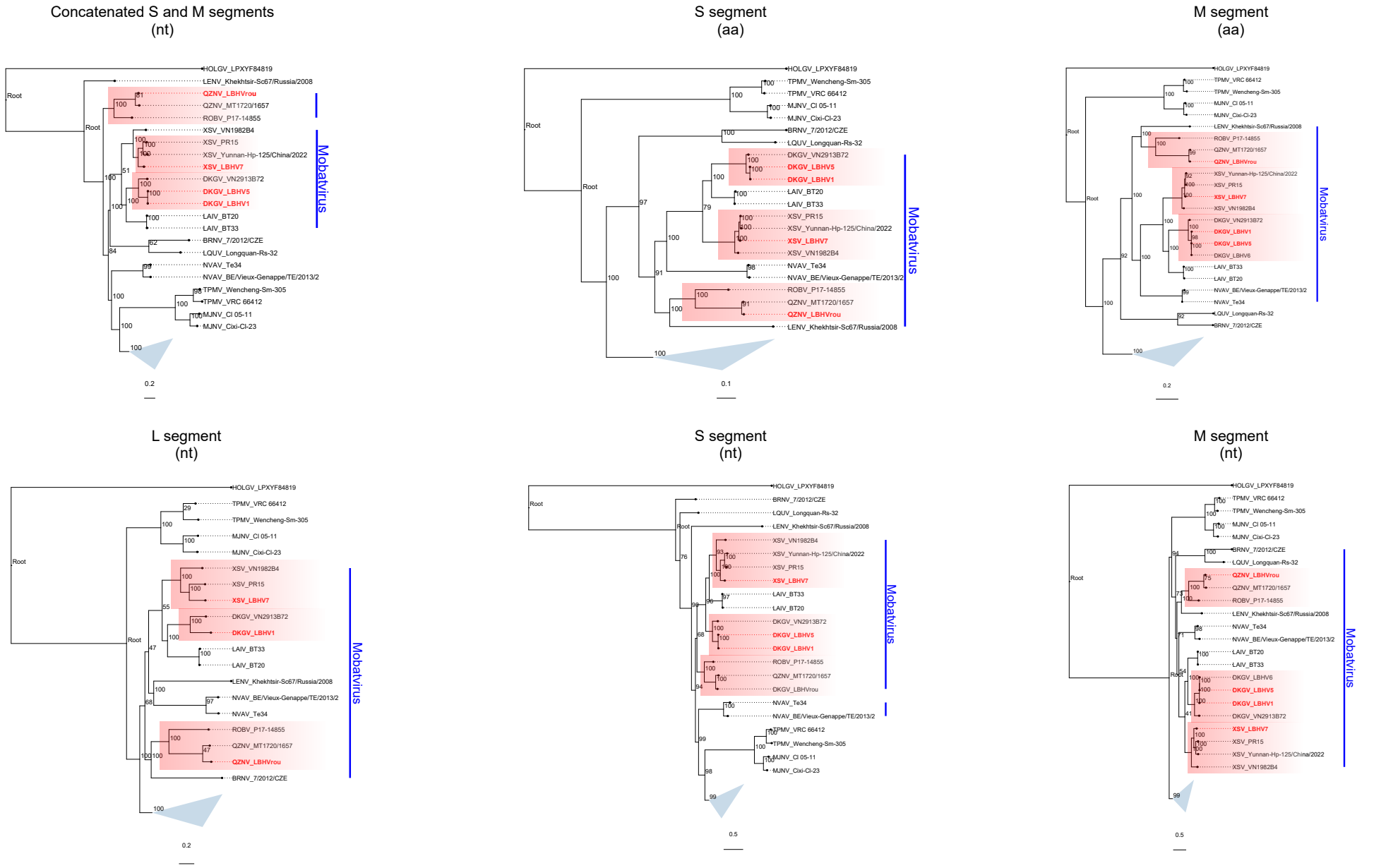

Supplement: Supplementary file 1 [file jgv-107-02333-s001.pdf]
